# Talc Use and Risk of Cancer: An Umbrella Review and Overlapped Analysis

**DOI:** 10.1002/cnr2.70705

**Published:** 2026-10-05

**Authors:** Frank Mayta‐Tovalino, Miguel Cabanillas‐Lazo, Carlos Quispe‐Vicuña, Fran Espinoza‐Carhuancho, Adrian V. Hernandez

**Affiliations:** ^1^ Vicerrectorado de Investigación Universidad San Ignacio de Loyola Lima Peru; ^2^ Grupo de Investigación NEMECS: Neurociencias, Metabolismo, Efectividad Clínica y Sanitaria Universidad Científica del Sur Lima Peru; ^3^ Faculty of Medicine Universidad Nacional Mayor de San Marcos Lima Peru; ^4^ Health Outcomes, Policy and Evidence Synthesis (HOPES) Group University of Connecticut School of Pharmacy Storrs Connecticut USA

## Abstract

**Background:**

This umbrella review aimed to systematically synthesize and critically appraise the available evidence from systematic reviews and meta‐analyses evaluating the association between talc exposure and cancer risk in humans.

**Methods:**

This umbrella review was conducted in accordance with the PRIOR guidelines and registered in PROSPERO. A comprehensive search of PubMed, Embase, Scopus, Web of Science, and Epistemonikos was performed up to October 2024 without language or date restrictions. Eligible studies were systematic reviews evaluating any type of talc exposure in humans. Study selection and data extraction were performed independently, and risk of bias was assessed using the AMSTAR‐2 tool.

**Results:**

Nine systematic reviews were included, eight combining cohort and case–control studies and one restricted to cohort studies. Cosmetic talc exposure was the most frequently investigated, with most reviews focused on ovarian cancer. Moderate overlap of study populations (9.2%) was identified, primarily related to Boon et al. Based on AMSTAR‐2, confidence in the evidence was moderate in one review and critically low in seven, with frequent methodological shortcomings such as lack of protocol registration and unclear study exclusions. For occupational exposure, elevated risks were observed for overall cancer (RR 1.21; 95% CI 1.03–1.42) and colorectal cancer (SIR 1.59; 95% CI 1.07–2.26), while no increased risk was found for other cancer types. Four of five systematic reviews evaluating cosmetic talc exposure and ovarian cancer reported a statistically significant association with increased ovarian cancer risk, whereas one review found no statistically significant association. However, confidence in the results of the reviews included was predominantly low or critically low, reflecting important methodological limitations, including concerns regarding risk‐of‐bias assessment and reporting. Overall, the evidence supporting an association between cosmetic talc exposure and ovarian cancer was of limited confidence.

**Conclusion:**

There is limited evidence from systematic reviews regarding the association of exposure to cosmetic talc with an increased risk of developing ovarian cancer. Therefore, the results of the systematic reviews should be regarded as suggestive, rather than definitive, and further studies are required to demonstrate a causal relationship.

## 1 Introduction

Cancer is defined as abnormal cell proliferation that leads to the invasion and spread of cancerous cells (metastasis) throughout the body. Many people across the globe die from cancer each year and cancer is among the top causes of fatalities worldwide; as of 2022 there were 20 million newly diagnosed cases and almost 10 million cancer fatalities [1]. Expected employment statistics for 2050 show a continued increase in numbers for developed countries, while developing countries will experience a greater percentage of deaths due to cancer [1, 2]. Cancer has a direct impact on individuals in terms of their health, as well as the quality of life of those affected, because of cancer‐related morbidity and the side effects of treatment [3]. Additionally, the cost associated with cancer includes direct costs associated with medical treatment of cancer, loss of work productivity due to cancer or when caring for other family members with cancer, and informal caregivers of those with cancer. These costs can create a very large financial burden on both cancer patients and their families, as well as the health care systems that provide services for these patients [4].

Talc is a mineral that forms naturally from the combination of magnesium and silica at high temperatures under pressure, and it can be found in many places around the globe. There are three ways humans meet talc: cosmetic contact from daily hygiene products [5], occupational contact through work in processing mines [6], and occupational and para‐occupational exposure has been documented in populations residing near talc mining sites and processing facilities. For decades, the potential association between talc exposure and respiratory conditions—including pneumoconiosis, granulomatous reactions, pulmonary fibrosis, and pulmonary hypertension—has remained a subject of ongoing debate [7].

There has been considerable interest in investigating the pathophysiological mechanism(s) of an association between the use of talc in the perineal area and ovarian cancer. One theory is that talc particles can travel through the genital tract and reach the ovaries, causing chronic inflammation and thus leading to an environment that promotes tumor growth [8]. Chronic inflammation is well documented as a contributing factor in the development of various cancers due to the release of cytokines and reactive oxygen species by inflammatory cells damaging DNA and stimulating cell proliferation [9]. Animal and human tissue studies have demonstrated that talc particles may promote proliferation and therefore may be carcinogenic [10]. Even though a conclusive cause and effect relationship has not yet been determined, the data available provide a strong basis for further exploration of the potential link between extended talc use and the development of ovarian cancer in women. Although several hypotheses have been proposed, direct evidence demonstrating particle migration to ovarian tissue in humans remains limited.

Several systematic reviews (SRs) have been conducted, considering the clinical and methodological heterogeneity of the included studies. Therefore, the aim of this umbrella review was to synthesize and critically evaluate evidence from SRs and meta‐analyses on talc exposure and cancer risk, including risk of bias (RoB) assessment and overlap analysis.

## 2 Methods

### 2.1 Study Design

The protocol for this umbrella review was registered in PROSPERO (registration number: CRD 42024575802). The study was reported according to the Preferred Reporting Items for Overviews of Reviews (PRIOR) [11].

### 2.2 Search Strategy

A systematic search was conducted in five large databases (PubMed, Embase, Scopus, Web of Science) for studies published until October 15, 2024. The search strategy was developed according to the requirements of the relevant database, while leaving methodological consistency as much as possible. Search strategies are outlined in Supplementary Material S1. No restrictions were placed based on geographical location, language, or publication date. The authors additionally checked references of included studies and selected additional articles that met the eligibility criteria.

### 2.3 Selection Criteria

Studies were eligible if they met the following criteria: (1) SRs, with or without meta‐analysis; (2) included human populations of any age or sex, including the general population or specific occupational or clinical populations; and (3) evaluated exposure to talc through any route, including cosmetic (e.g., perineal or personal care products), occupational (e.g., mining, milling, manufacturing, or industrial use), environmental, or other documented sources, in relation to the risk of any type of cancer. We excluded primary observational or interventional studies, animal or in vitro studies, case reports, narrative reviews, conference abstracts, editorials, letters, and commentaries. Gray literature sources and the reference lists of the included SRs were also screened to identify additional eligible studies.

### 2.4 Study Selection

The findings of the electronic database search were initially imported into EndNote X9 and duplicates were identified and removed. The remaining records were then exported to Rayyan (https://rayyan.qcri.org/) for independent screening by M.C.‐L. and C.Q.‐V. of titles and abstracts; discrepancies were resolved with the help of a third author (F.M.‐T.). M.C.‐L. and C.Q.‐V. worked independently to determine whether the full text of included studies met the specified and predetermined inclusion criteria. Any differences were resolved through consensus.

### 2.5 Data Extraction

Two authors (M.C.‐L. and C.Q.‐V.) independently extracted the data using a prespecified data extraction form; disagreements were resolved by consensus and, ultimately, by a third author (F.M.‐T.). We extracted the following information: study title, first author, year of publication, number of participants, sex, age, sampling time, type of talc exposure, crude and adjusted measures of association, and type of cancer. If additional data were required, we contacted the corresponding author by email to request further information. Supplementary Material S2.

### 2.6 RoB Assessment

Two independent reviewers (M.C.‐L. and C.Q.‐V.) assessed the RoB in the included SRs using the AMSTAR‐2 (Assessing the Methodological Quality of Systematic Reviews 2) tool [12], resolving disagreement by consensus and involving a third author (F.M.T.) in the process. The AMSTAR‐2 is composed of 16 items that assess methodological quality; each item is scored using four different options: “Yes,” “Partially Yes,” “No,” and “Not applicable to the meta‐analysis.” SRa were assigned to “high confidence in the results of the review” rating: the majority of items were scored as “Yes,” or more were “Yes” than “No” (even if fully met); “moderate confidence” rating: when several noncritical weaknesses were found, and we felt it was unlikely (given the number) that affected an overall conclusion; “low confidence” rating: one or more critical flaws were found that negatively affected the reliability of findings in at least one (but maybe several overall) major domain; and “critically low confidence” when there were multiple critical flaws in a number of major domains, and therefore the review cannot be trusted.

### 2.7 Overlap Analysis

An overlap analysis was performed because some reviews used some studies at the same time. The primary studies included in each SR are listed in a table (Supplementary Material S3). Overlap was then calculated as the corrected area covered according to the formula: ((*N* − *b*)/(*bc* − *b*)) × 100 (*N* is the total number of primary studies, *b* is the number of primary studies excluding overlapping studies, and c: number of SRs). We interpreted the results in the following categories: slight overlap (0%–5%), moderate (6%–10%), high (11%–15%), and very high (> 15%) [13].

## 3 Results

### 3.1 Study Selection

Through our systematic search, we identified 643 studies. We removed duplicates and examined 290 studies by title and abstract. The main reasons for exclusion were other populations (*n* = 2), other outcomes (*n* = 2), absence of systematic search (*n* = 2), and other exposure (*n* = 1). Finally, we included nine studies for analysis (Figure 1). The complete list of excluded articles is provided in Supplementary Material S2.

**FIGURE 1:**
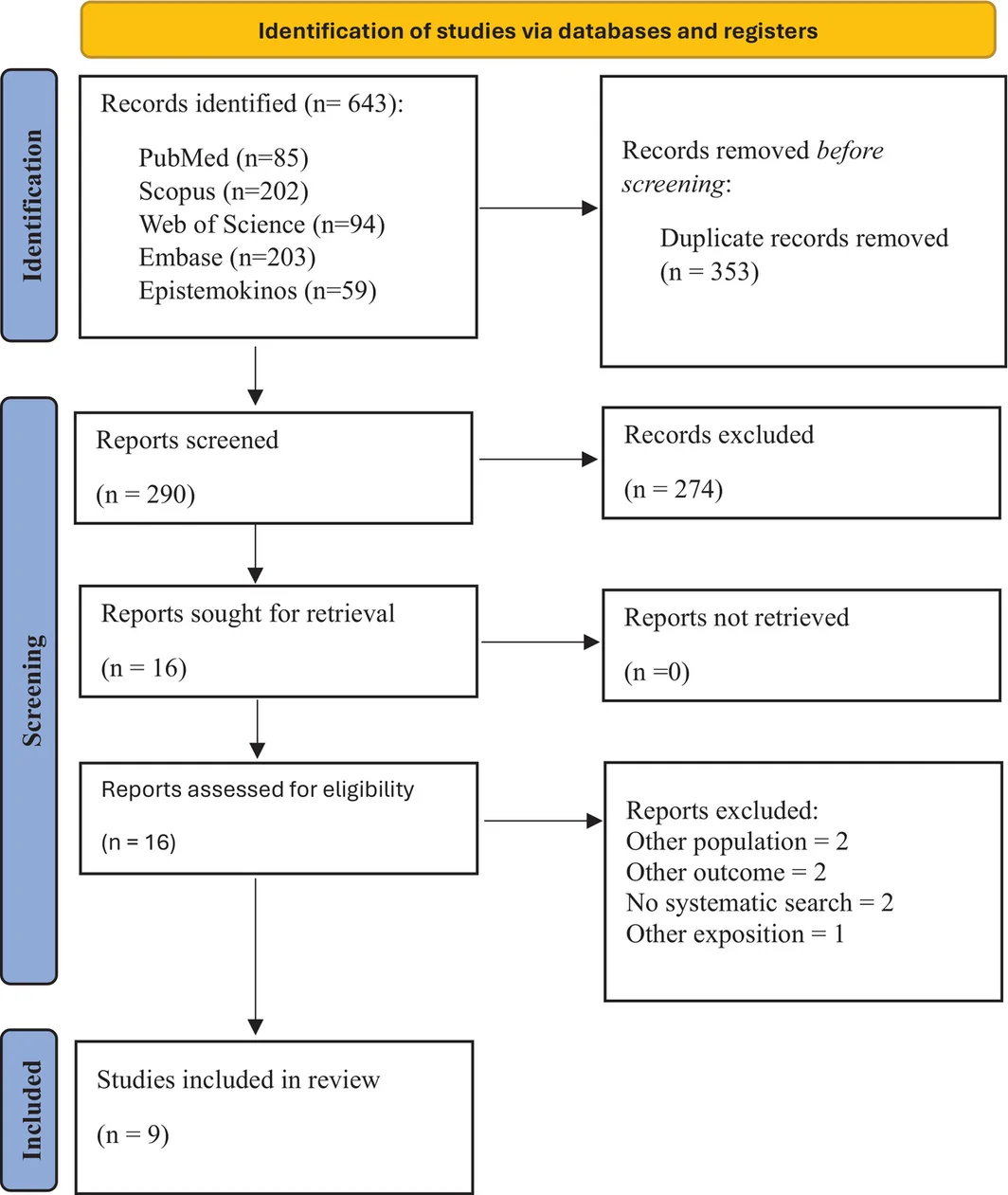
PRISMA flowchart of included studies.

### 3.2 Characteristics of the Included Studies

Nine SRs included [1, 2, 3, 4, 5, 6, 7, 8, 9] both cohort and case–control studies, while one SR only included cohorts. The most studied type of cancer was ovarian cancer, while exposure through cosmetics was the most frequent (six SRs). Table 1 provides a summary of the characteristics of the included studies (Supplementary Material S5) [14, 15, 16, 17, 18, 19, 20, 21, 22].

**TABLE 1:** Characteristics included systematic reviews.

References	Study design of studies included	Study number	Population	Sample size (*n*)	Gender	Type of exposition	Type of cancer	Risk of bias	Evaluation of certainty of evidence	Funding
Woolen [14]	Cohort and case–control studies	11	General population	73 418	Female	Cosmetic	Ovarian cancer	Newcastle Ottawa Scale	No	Self‐funded
Penninkilampi and Eslick [15]	Cohort and case–control studies	27	General population	NR	Female	Cosmetic	Ovarian cancer	Newcastle Ottawa Scale	No	No reported
Lewis Ryan [16]	Cohort and case–control studies	12	Barbers, hairdressers, and cosmetologists	NR	Male/female	Occupational	Mesothelioma	No	No	Benchmark Risk Group
Kadry Taher [17]	Cohort and case–control studies	30	General population	NR	Female	Cosmetic	Ovarian cancer	Newcastle Ottawa Scale	No	Risk Sciences International
Huncharek [18]	Cohort and case–control studies	9	Women that use of talc‐dusted contraceptive diaphragms.	5889	Female	Cosmetic	Ovarian cancer	No	No	Johnson and Johnson Company
Boon et al. [19]	Cohort and case–control studies	77	General and specific population	NR	Male/female	Occupational and cosmetic	Reproductive tract cancer, respiratory tract cancer, gastrointestinal cancer, urinary system cancer, lymphohematopoietic cancer, male cancer, CNS cancer, skin cancer	Made by the authors	Yes, through Bradford‐Hill considerations	Essential Minerals Association
Finley et al. [20]	Cohort and case–control studies	3	Talc miners and millers	4443	Male/female	Occupational	Mesothelioma	No	No	Self‐funded
Chang [21]	Cohort studies	12	Workers with occupational talc exposure	NR	Male/female	Occupational	Stomach cancer	Newcastle Ottawa Scale	No	Ministry of Science and Technology of Taiwan
Berge [22]	Cohort and case–control studies	28	General population	NR	Female	Cosmetic	Ovarian cancer	Newcastle Ottawa Scale	No	Self‐funded

### 3.3 Overlap Assessment

Among the SRs found, there was moderate overlap (9.2%) of primary studies. This is mainly because the Boon et al. study covers a large percentage of the articles (Supplementary Material S3).

### 3.4 RoB

Only one SR was judged to be of moderate confidence, while seven SRs were judged to be critically low confidence (Supplementary Material S4). All except the Boon et al. [15]. SR lacked the critical domain related to reporting the reasons for exclusion. Six SRs did not include details about an explicit statement that the review methods were established prior to conducting the review.

### 3.5 Cosmetic Use of Talc

Four out of five SRs reported statistically significant associations between cosmetic use of talc and the risk of ovarian cancer. Boon et al. reported an increased risk in one of two studies (hazard ratio [HR]: 2.13; 95% CI 1.54–2.94). No added risk was reported for cervical and uterine cancer.

### 3.6 Occupational Risk of Talc

One SR reported that occupational exposure to talc increased the risk of cancer (relative risk [RR]: 1.21; 95% CI: 1.03–1.42). In addition, an increased risk of colorectal cancer was reported (standardized incidence rate ratio [SIR]: 1.59; 95% CI: 1.07–2.26). However, there was no statistically significant association for the risk of pleural, peritoneal, lung, bladder, kidney, and prostate cancer.

## 4 Discussion

This umbrella review synthesized the available evidence from SRs and meta‐analyses evaluating the association between talc exposure and cancer risk. Overall, four of five SRs evaluating perineal cosmetic talc exposure and ovarian cancer reported statistically significant associations, whereas one review found no statistically significant association. Evidence regarding occupational talc exposure suggested possible associations with certain gastrointestinal cancers; however, these findings remain inconclusive. The interpretation of these findings requires caution because of methodological heterogeneity, the predominance of retrospective observational designs, potential limitations and biases in the underlying primary studies, and the overall low to critically low confidence in the results of the SRs.

The findings are consistent with previous meta‐analyses that have reported small relative increases in risk. For example, a meta‐analysis that included primarily case–control studies reported a significant association between perineal talc use and ovarian cancer (OR 1.31; 95% CI 1.24–1.39), with a higher risk at high cumulative exposures (> 3600 applications: OR 1.42; 95% CI 1.25–1.61) and differences according to tumor histological subtype [2]. Consistently, another meta‐analysis identified a weaker overall association (RR 1.22; 95% CI 1.13–1.30), with significant heterogeneity observed across study designs and no association in cohort studies (RR 1.02; 95% CI 0.85–1.20), with evidence limited primarily to serous carcinoma (RR 1.24; 95% CI 1.15–1.34) [9]. The results of the present review replicate this general pattern, in which positive associations are predominantly concentrated in retrospective studies, while prospective studies show estimates closer to null.

The discrepancy between epidemiological designs is a central element in causal interpretation. Case–control studies are particularly susceptible to recall bias and differential classification of exposure, especially when information is based on retrospective self‐reporting of personal hygiene practices. Comprehensive SRs have indicated that consistent associations with ovarian cancer may be influenced by these methodological biases and residual confounding factors rather than by a direct biological effect [6]. In fact, reviews integrating multiple lines of epidemiological evidence have concluded that there is insufficient support for a causal relationship between talc exposure and cancer in humans [6]. Our findings, incorporating critical quality assessment and overlap analysis, reinforce the need for cautious interpretation of the observed association.

Histological subtype evidence is also an important source of information. Both Penninkilampi and Berge found mostly associations with invasive serous carcinoma, and only a small portion of the other subtypes [15, 22]. The nature of the observed associations may imply a mechanism with biological plausibility, but they also may reflect limitations in the statistical assessment of the studies, diagnostic heterogeneity, or a differential bias between the studies. The reason for the proposed mechanism of action (upward migration of specific particles and chronic inflammation) remains largely unproven in humans.

Other reviews indicate negative findings. For instance, a later meta‐analysis evaluating talc‐containing birth control diaphragms did not find conclusive evidence of risk (RR = 1.03; 95% CI 0.80–1.37). This indicates that there is no association in the context of this method of talc exposure [5]. Similarly, the reviews that combine epidemiological, mechanistic, and experimental evidence collectively classify the evidence as “suggestive” of no association between the use of talc on female genitalia and cancer of the female reproductive system at levels of talc exposure that are relevant to humans [10]. Therefore, these findings indicate continued uncertainty and the need for studies that objectively measure talc exposure levels.

There is less evidence available on occupational exposures than there is on other exposures; however, there is evidence to indicate that occupational exposure may increase the risk of certain gastrointestinal cancers. Specifically, one study conducted a meta‐analysis of occupational cohorts and found a statistically significant association between talc exposure and gastric cancer (RR 1.21; 95% CI 1.03–1.42). This association was no longer statistically significant when limited to only that portion of the talc products that did not contain asbestiform fibers (RR 1.26; 95% CI 0.97–1.63) [8]. When considering that historical contamination with asbestos may be a confounding variable when conducting an occupational study, it is important to note that it is difficult to establish the causation of talc and gastric cancer.

Considering the evidence hierarchy, perineal talc use is consistent with existing comprehensive reviews of ovarian cancer risk factors, where it seems to be associated with a lesser relative increase in risk than is seen with genetic, hormonal, or metabolic determinants [15]. Therefore, it is likely that even under a causal hypothesis, the overall contribution of talc use to the development of ovarian cancer among the general population would be small.

SRs and meta‐analyses have continued to find a small to moderate increase in the risk of developing ovarian cancer for women who use talcum powder for cosmetic purposes. However, the prospective nature of only a few studies has been found to have inconsistent estimates. The most recent SR conducted by Boon et al. [19] was only able to identify one study with a prospective design that showed a statistically significant relationship between talc use and ovarian cancer and therefore did not provide a pooled estimate. Therefore, while earlier SRs provided consistent information regarding this risk, they should not be seen as providing definitive evidence of a link between talc use and ovarian cancer. Our umbrella review can only conclude that all available data indicate a potential association between the use of talcum powder and the development of ovarian cancer; however, the certainty of this association is currently limited due to the limited number of studies that used a prospective design and the low to critically low methodological confidence of almost all of the included SRs per AMSTAR 2.

### 4.1 Study Limitations

An important methodological finding of this review was the predominantly critically low quality of the reviews included. The most frequent deficiencies included the absence of registered protocols, lack of justification for exclusions, and incomplete assessment of RoB, which reduces confidence in the summary estimates. In addition, the overlap analysis showed a moderate degree of redundancy between reviews, indicating partial dependence on primary evidence and possible overestimation of the consistency of the findings [23, 24].

The assessment of the causality must consider multiple epidemiological dimensions. The observed effect size is small (estimates generally between 1.2 and 1.3), consistency depends on study design, dose–response evidence is limited, and biological plausibility is not confirmed in humans. Taking together, these elements suggest that the current evidence is insufficient to establish a causal relationship with high certainty, particularly for cosmetic exposures.

There is a very important issue regarding the distinction between talc without asbestos fibers and talc that has been historically contaminated with asbestos fibers, especially in the workplace. Although there were SRs that included this issue, primary studies could not classify exposures consistently; therefore, it was impossible to exclude residual exposure misclassification. Furthermore, there is increased importance for this distinction due to the recent IARC classification of talc without asbestos as likely carcinogenic to humans (Group 2A). However, there is much controversy about how the decision was interpreted scientifically. For example, one critical review suggested that the classification of talc without asbestos as a carcinogen was primarily due to changes in the evaluation framework rather than new evidence from epidemiology or laboratory studies. Furthermore, it highlighted ongoing uncertainty regarding mineralogical characterization, how mechanistic data are interpreted, and the total weight of evidence for carcinogenicity. Thus, the associations documented in this umbrella review (particularly for occupational exposure) must be interpreted with caution until more definitive studies can separate talc without asbestos from talc contaminated with asbestos using similar exposure assessment techniques [25, 26].

There is another important consideration when evaluating human exposures to talc due to the way talc is used specifically for cosmetic products applied to the Perineum and in certain occupational environments where workers are around talc routinely; however, talc also has very broad commercial and industrial uses and is found in many different products (cosmetics, pharmaceuticals, food additives (in some countries), paper, plastics, ceramics, paints, and rubber) with multiple potential sources and pathways of human exposure with varying frequencies, intensities and routes of exposure. With this point being made, it should be noted that the SRs included in the current umbrella have primarily focused on Perineal Cosmetic use and Occupational exposure, with limited or no information available for the vast majority of alternate paths for talc exposure. Therefore, the findings presented here should not be interpreted as indicative of the health risks associated with the entire range of potential exposure to talc; additionally, future studies examining the health effects of talc on humans should include a comprehensive review of those potential exposures, using standardized methods for assessing and evaluating exposure to talc [27, 28].

Occupational evidence should be interpreted with caution, as most of the studies available regarding talc exposure are based on historical cohorts with little to no characterization of the talc exposure (some studies had substantial differences in the level of the talc exposure) and also did not have good information regarding potential co‐exposures (i.e., asbestos or other workplace carcinogens). In the meta‐analysis of occupational studies included in this umbrella review, the association between talc exposure and stomach cancer became attenuated and no longer statistically significant when the analyses were restricted to checking for talc with no asbestiform fibers, suggesting that it is possible that residual confounding from asbestos contamination is likely not fully ruled out in the reported studies. However, there are also differences between the various cohorts regarding the type of industry that the workers were employed in, the intensity of the exposure, the length of time of exposure, the smoking practices of the workers in the various studies and the other substances that may have also contributed to the workers' exposure in the workplace. Therefore, the occupational evidence available should be interpreted as being suggestive, rather than conclusive. Future studies should incorporate the use of standard mineralogical characterization of talc and standardized assessment of other substances the worker may have been exposed to, in order to obtain the most accurate causal inferences.

Additionally, determining the potential role of mineral contaminants in cancer risk also requires interpreting the data related to fiber morphology and mineralogical composition of the talc contaminating minerals. Many historical studies related to occupational health have not included complete documentation regarding the morphology of the asbestos and non‐asbestos (asbestiform and elongated) talc deposits, making it difficult to differentiate between the two types of minerals as potentially harmful to human health. Therefore, as a result, the complete understanding of how the talc contaminants may have been involved in causing increased incidence of cancer is not yet known and future studies need to consider standardized methodology for conducting mineral analyses of talc contaminants. By using contemporary analysis methods, future studies will provide a much more comprehensive understanding of how talc contaminants may have impacted the health of an occupational group. This umbrella review has inherent design limitations, including dependence on the quality of previous reviews, heterogeneity in the classification of exposures, and absence of second‐order meta‐analyses due to study overlap. However, the incorporation of overlap analysis and systematic evaluation of methodological quality is a significant strength.

The current evidence does not provide sufficient basis for a determination of whether or not talc that is free of asbestos or other impurities is carcinogenic; the epidemiologic studies reviewed were insufficiently designed to adequately control for or evaluate possible contamination from other minerals. As such, it is possible that the residual confounding or the misclassification of exposure within these studies may be a source of error. The association between occupational exposure to talc and the increased incidence of colorectal cancer should also be interpreted cautiously, as the evidence in the currently available literature does not support a deterministic relationship between exposure to talc and development of colorectal cancer. As there remains a lack of information regarding the reason for the increased incidence of colorectal cancer, it is recommended that additional studies be performed that are designed with appropriate mineralogical characterization and rigorously evaluate potential occupational co‐exposures to talc. Another limitation is that we didn't evaluate the possibility of bias in each of the single primary studies contained within the SRs. So, potential biases, especially recall bias and differential exposure misclassification related to case–control studies investigating the relationship between the use of cosmetic talc and ovarian cancer, could have impacted the outcomes reported by the included studies and should be accounted for when considering our results.

## 5 Conclusions

The available evidence is insufficient to establish whether cosmetic talc exposure increases the risk of ovarian cancer. Although some systematic studies show a statistically significant association between cosmetic talc exposure and the risk of developing ovarian cancer, the studies themselves consistently show a low or very low confidence in their findings, largely due to serious limitations in their methodology, variability across different types of studies, the overlap of the SRs, and the possible biases of the observational study data on which they were based. A similar lack of confidence exists for the evidence regarding the relationship between workplace exposure to talc and gastrointestinal cancers. Due to the currently available evidence, we cannot conclude that talcum powder was a cause of cancer in humans. Future prospective studies incorporating high‐quality methodologies to evaluate talc and any other substances that may be present in talc, plus proper controls for confounding variables, will help determine whether or not there is a connection between talcum powder exposure and cancer. Additionally, methodologically rigorous SRs will provide more clarity regarding the associations discussed.

## Author Contributions

**Frank Mayta‐Tovalino:** conceptualization, writing – review and editing, formal analysis, supervision, project administration, funding acquisition. **Miguel Cabanillas‐Lazo:** conceptualization, investigation, methodology, software, formal analysis, writing – original draft, writing – review and editing. **Carlos Quispe‐Vicuña:** conceptualization, investigation, methodology, writing – original draft, writing – review and editing, software. **Fran Espinoza‐Carhuancho:** conceptualization, investigation, writing – original draft. **Adrian V. Hernandez:** supervision, formal analysis, visualization, project administration, writing – review and editing, conceptualization, methodology.

## Funding

The authors have nothing to report.

## Ethics Statement

The authors have nothing to report.

## Conflicts of Interest

The authors declare no conflicts of interest.

## Supporting information


**Supplementary Material S1.** Search strategy.
**Supplementary Material S2**. Excluded studies.
**Supplementary Material S3**. Overlap measurement.
**Supplementary Material S4**. AMSTAR‐2 ratings for included systematic review displayed as risk of bias.
**Supplementary Material S5**. Results of the included systematic reviews according to outcomes (*n* = 9).

## Data Availability

All the data presented in this manuscript are available from the corresponding authors and can be requested from the corresponding author.
