# Supplementary material for: Talc Use and Risk of Cancer: An Umbrella Review and Overlapped Analysis
Source: Cancer Rep (Hoboken). 2026 Oct 5;9(10):e70705. doi: 10.1002/cnr2.70705 (PMC13638487; doi:10.1002/cnr2.70705)
Supplement: Supplementary file 1 [file CNR2-9-e70705-s001.docx]

**Talc use and risk of cancer: An umbrella review and overlapped analysis**

**Supplementary material S1. Search strategy**

| 1 | **Pubmed:**  #1: (talc[mh] OR talc*[tiab] OR powder*[TIAB] OR steritalc*[TIAB] OR sclerosol[tiab] OR powders[mh])  #2: (neoplasms[mh] OR Tumor*[tiab] OR Neoplas*[tiab] OR Cancer*[tiab] OR Malignan*[tiab] OR Tumour*[tiab] OR Oncolog*[tiab])  #3: (Systematic Review [Publication Type] OR “Systematic Reviews as Topic”[MH] OR “Systematic Review*”[tiab] OR Meta-Analysis [Publication Type] OR “Meta-Analysis as Topic”[MH] OR Meta-Analys*[tiab] OR “Meta analys*”[tiab])  #4: #1 AND #2 AND #3 |
| --- | --- |
| 2 | **Scopus:**  #1: TITLE-ABS-KEY(talc* OR powder* OR steritalc* OR sclerosol)  #2: TITLE-ABS-KEY(Tumor* OR Neoplas* OR Cancer* OR Malignan* OR Tumour* OR Oncolog*)  #3: TITLE-ABS-KEY(“Systematic Review*” OR Meta-Analys* OR “Meta analys*”)  #4: #1 AND #2 AND #3 |
| 3 | **Web of Science:**  #1: TI=(talc* OR powder* OR steritalc* OR sclerosol) OR AB=(talc* OR powder* OR steritalc* OR sclerosol) OR KP=(talc* OR powder* OR steritalc* OR sclerosol) OR AK=(talc* OR powder* OR steritalc* OR sclerosol)  #2: TI=(Tumor* OR Neoplas* OR Cancer* OR Malignan* OR Tumour* OR Oncolog*) OR AB=(Tumor* OR Neoplas* OR Cancer* OR Malignan* OR Tumour* OR Oncolog*) OR AK=(Tumor* OR Neoplas* OR Cancer* OR Malignan* OR Tumour* OR Oncolog*) OR KP=(Tumor* OR Neoplas* OR Cancer* OR Malignan* OR Tumour* OR Oncolog*)  #3: TI=(“Systematic Review*” OR Meta-Analys* OR “Meta analys*”) OR AB=(“Systematic Review*” OR Meta-Analys* OR “Meta analys*”) OR KP=(“Systematic Review*” OR Meta-Analys* OR “Meta analys*”) OR AK=(“Systematic Review*” OR Meta-Analys* OR “Meta analys*”)  #4: #1 AND #2 AND #3 |
| 4 | **Embase:**  ('talc'/exp OR 'sclerosol' OR 'siliconized talcum' OR 'steritalc' OR 'talc' OR 'talc powder, sterile' OR 'talcum' OR 'talcum powder' OR 'powder'/exp OR 'face powder' OR 'powder' OR 'powder analysis' OR 'powders') AND ('malignant neoplasm'/exp OR 'cancer' OR 'cancers' OR 'malignant neoplasia' OR 'malignant neoplasm' OR 'malignant neoplastic disease' OR 'malignant tumor' OR 'malignant tumour' OR 'neoplasia, malignant' OR 'neoplasmic malignancy' OR 'neoplastic malignancy' OR 'oncologic malignancy' OR 'oncological malignancy' OR 'tumor, malignant' OR 'tumoral malignancy' OR 'tumorous malignancy' OR 'tumour, malignant') AND ('systematic review'/exp OR 'review, systematic' OR 'systematic review' OR 'systematic review (topic)'/exp OR 'systematic review (topic)' OR 'systematic reviews' OR 'systematic reviews as topic' OR 'meta analysis'/exp OR 'analysis, meta' OR 'meta analysis' OR 'meta-analysis' OR 'metaanalysis' OR 'meta analysis (topic)'/exp OR 'meta analysis (topic)' OR 'meta-analysis as topic' OR 'metaanalyses') |
| 5 | **Epistemonikos**  #1: (talc* OR powder* OR steritalc* OR sclerosol)  #2: (Tumor* OR Neoplas* OR Cancer* OR Malignan* OR Tumour* OR Oncolog*)  #3: (“Systematic Review*” OR Meta-Analys* OR “Meta analys*”)  #4: #1 AND #2 AND #3 |

**Supplementary material S2. Excluded studies**

| **Title** | **Author** | **Reason for exclusion** |
| --- | --- | --- |
| Systematic review of the scientific evidence of the pulmonary carcinogenicity of talc | Lynch, Heather | Specifically, it focused exclusively on the pulmonary carcinogenicity of inhaled talc, incorporated both human and animal evidence within the same evidence synthesis framework, |
| Systematic review of the association between talc and female reproductive tract cancers | Lynch, Heather | This review was excluded because it was designed as an integrated weight-of-evidence assessment combining epidemiological, animal, and mechanistic evidence, whereas our umbrella review included only systematic reviews synthesizing epidemiological evidence from human populations. |
| Genital Powder Use and Risk of Ovarian Cancer: A Pooled Analysis of 8,525 Cases and 9,859 Controls | Terry, Kathryn | No systematic search |
| Malignant pleural effusions and the role of talc poudrage and talc slurry: a systematic review and meta-analysis | Mummadi, Srinivas | Other exposition |
| Risk of Mortality from Respiratory Malignant and Non-Malignant Diseases among Talc Miners and Millers: A Systematic Review and Meta-Analysis | Ciocan, Catalina | Although this review included lung cancer mortality as a secondary outcome, its primary objective was to evaluate mortality from respiratory diseases in occupational cohorts rather than the association between talc exposure and cancer risk. Therefore, it was considered outside the scope of our umbrella review, which was restricted to systematic reviews assessing cancer incidence or risk as the primary outcome. |
| Talc, body powder, and ovarian cancer: A summary of the epidemiologic evidence | Wentzensen, Nicolas | No systematic search |
| Association between pelvic inflammatory disease and risk of ovarian cancer: An updated meta-analysis | Piao, Jinlan | Other outcome |

**Supplementary material S3. Overlap measurement**

|  | **Lewis (2023)** | **Woolen (2022)** | **Finley (2017)** | **Boon (2024)** | | | **Berge (2017)** | | **Chang (2020)** | **Huncharek (2007)** | **Penninkilampi (2018)** | **Kadry Taher (2019)** |
| --- | --- | --- | --- | --- | --- | --- | --- | --- | --- | --- | --- | --- |
| Andersen 1999 |  |  |  |  | | |  | |  |  |  |  |
| Plato 2016 |  |  |  |  | | |  | |  |  |  |  |
| Pukkala 2009 |  |  |  |  | | |  | |  |  |  |  |
| Bouchardy 2002 |  |  |  |  | | |  | |  |  |  |  |
| Cocco 1999 |  |  |  |  | | |  | |  |  |  |  |
| Migliori 2022 |  |  |  |  | | |  | |  |  |  |  |
| Rodelsperger 2001 |  |  |  |  | | |  | |  |  |  |  |
| Swanson 1995 |  |  |  |  | | |  | |  |  |  |  |
| Teschke 1997 |  |  |  |  | | |  | |  |  |  |  |
| McElvenny 2005 |  |  |  |  | | |  | |  |  |  |  |
| Petersen 1980 |  |  |  |  | | |  | |  |  |  |  |
| Simpson 1999 |  |  |  |  | | |  | |  |  |  |  |
| Booth 1989 |  |  |  |  | | |  | |  |  |  |  |
| Chang 1997 |  |  |  |  | | |  | |  |  |  |  |
| Cook 1997 |  |  |  |  | | |  | |  |  |  |  |
| Cramer 2016 |  |  |  |  | | |  | |  |  |  |  |
| Harlow 1992 |  |  |  |  | | |  | |  |  |  |  |
| Mills 2004 |  |  |  |  | | |  | |  |  |  |  |
| Rosenblatt 2011 |  |  |  |  | | |  | |  |  |  |  |
| Schildkraut 2016 |  |  |  |  | | |  | |  |  |  |  |
| Whittemore 1988 |  |  |  |  | | |  | |  |  |  |  |
| Wu 2009 |  |  |  |  | | |  | |  |  |  |  |
| O’Brien 2020 |  |  |  |  | | |  | |  |  |  |  |
| Coggiola 2003 |  |  |  |  | | |  | |  |  |  |  |
| Wergeland 1990 |  |  |  |  | | |  | |  |  |  |  |
| Wild A 2002 |  |  |  |  | | |  | |  |  |  |  |
| Wild F 2002 |  |  |  |  | | |  | |  |  |  |  |
| Brown 1990 |  |  |  |  | | |  | |  |  |  |  |
| Bulbulyan 1999 |  |  |  |  | | |  | |  |  |  |  |
| Chang 2019 |  |  |  |  | | |  | |  |  |  |  |
| Chen 1992 |  |  |  |  | | |  | |  |  |  |  |
| Chiazze 1993 |  |  |  |  | | |  | |  |  |  |  |
| Ciocan 2022 |  |  |  |  | | |  | |  |  |  |  |
| Cramer 1982 |  |  |  |  | | |  | |  |  |  |  |
| Cramer 1995 |  |  |  |  | | |  | |  |  |  |  |
| Cramer 1999 |  |  |  |  | | |  | |  |  |  |  |
| Crawford 2012 |  |  |  |  | | |  | |  |  |  |  |
| Davis 2021 |  |  |  |  | | |  | |  |  |  |  |
| Fordyce 2019 |  |  |  |  | | |  | |  |  |  |  |
| Gabriel 2019 |  |  |  |  | | |  | |  |  |  |  |
| Gamble 2013 |  |  |  |  | | |  | |  |  |  |  |
| Gates 2008 |  |  |  |  | | |  | |  |  |  |  |
| Gates 2010 |  |  |  |  | | |  | |  |  |  |  |
| Gertig 2000 |  |  |  |  | | |  | |  |  |  |  |
| Godard 1998 |  |  |  |  | | |  | |  |  |  |  |
| Gonzales 2016 |  |  |  |  | | |  | |  |  |  |  |
| Green 1997 |  |  |  |  | | |  | |  |  |  |  |
| Harlow 1989 |  |  |  |  | | |  | |  |  |  |  |
| Hartge 1983 |  |  |  |  | | |  | |  |  |  |  |
| Hartge 1994 |  |  |  |  | | |  | |  |  |  |  |
| Honda 2002 |  |  |  |  | | |  | |  |  |  |  |
| Houghton 2014 |  |  |  |  | | |  | |  |  |  |  |
| Ierardi 2020 |  |  |  |  | | |  | |  |  |  |  |
| Ierardi 2022 |  |  |  |  | | |  | |  |  |  |  |
| Jordan 2007 |  |  |  |  | | |  | |  |  |  |  |
| Karageorgi 2010 |  |  |  |  | | |  | |  |  |  |  |
| Katsnelson 1979 |  |  |  |  | | |  | |  |  |  |  |
| Ke 1999 |  |  |  |  | | |  | |  |  |  |  |
| Kurta 2012 |  |  |  |  | | |  | |  |  |  |  |
| Lamm 1988 |  |  |  |  | | |  | |  |  |  |  |
| Langseth 2004 |  |  |  |  | | |  | |  |  |  |  |
| Leung 2023 |  |  |  |  | | |  | |  |  |  |  |
| Merritt 2008 |  |  |  |  | | |  | |  |  |  |  |
| Moorman 2009 |  |  |  |  | | |  | |  |  |  |  |
| Negri 1989 |  |  |  |  | | |  | |  |  |  |  |
| Neill 2012 |  |  |  |  | | |  | |  |  |  |  |
| Ness 2000 |  |  |  |  | | |  | |  |  |  |  |
| Nielsen 1994 |  |  |  |  | | |  | |  |  |  |  |
| O’Brien 2019 |  |  |  |  | | |  | |  |  |  |  |
| O’Brien 2021a |  |  |  |  | | |  | |  |  |  |  |
| O’Brien 2021b |  |  |  |  | | |  | |  |  |  |  |
| Phung 2022 |  |  |  |  | | |  | |  |  |  |  |
| Pira 2017 |  |  |  |  | | |  | |  |  |  |  |
| Purdie 1995 |  |  |  |  | | |  | |  |  |  |  |
| Ramanakumar 2008 |  |  |  |  | | |  | |  |  |  |  |
| Rosenblatt 1992 |  |  |  |  | | |  | |  |  |  |  |
| Rubino 1976 |  |  |  |  | | |  | |  |  |  |  |
| Rubino 1979 |  |  |  |  | | |  | |  |  |  |  |
| Selevan 1979 |  |  |  |  | | |  | |  |  |  |  |
| Stille 1982 |  |  |  |  | | |  | |  |  |  |  |
| Straif 2000 |  |  |  |  | | |  | |  |  |  |  |
| Terry 2013 |  |  |  |  | | |  | |  |  |  |  |
| Thomas 1990 |  |  |  |  | | |  | |  |  |  |  |
| Thomas 1987 |  |  |  |  | | |  | |  |  |  |  |
| Tzonou 1993 |  |  |  |  | | |  | |  |  |  |  |
| Urban 2015 |  |  |  |  | | |  | |  |  |  |  |
| Wergeland 1990 |  |  |  |  | | |  | |  |  |  |  |
| Wergeland 2017 |  |  |  |  | | |  | |  |  |  |  |
| Wong 1999 |  |  |  |  | | |  | |  |  |  |  |
| Wu 2015 |  |  |  |  | | |  | |  |  |  |  |
| Zhang 1989 |  |  |  |  | | |  | |  |  |  |  |
| Goodman 2008 |  |  |  |  | | |  | |  |  |  |  |
| Lo-Ciganic 2012 |  |  |  |  | | |  | |  |  |  |  |
| Fu 1992 |  |  |  |  | | |  | |  |  |  |  |
| Li 1998 |  |  |  |  | | |  | |  |  |  |  |
| McLean 2006 |  |  |  |  | | |  | |  |  |  |  |
| Nie 1992 |  |  |  |  | | |  | |  |  |  |  |
| Stern 2001 |  |  |  |  | | |  | |  |  |  |  |
| Shushan 1996 |  |  |  |  | | |  | |  |  |  |  |
|  |  |  |  | |  |  | |  |  |  |  |  |

**Supplementary material S4. AMSTAR-2 ratings for included systematic review displayed as risk of bias**

| **Domain** | **Chang (2018)** | **Huncharek (2007)** | **Lewis (2023)** | **Woolen (2022)** | **Finley (2017)** | **Boon (2024)** | **Berge (2017)** | **Kadry Taher (2019)** | **Penninkilampi (2018)** |
| --- | --- | --- | --- | --- | --- | --- | --- | --- | --- |
| Did the research questions and inclusion criteria for the review include the components of PICO? | 1 | 1 | 1 | 1 | 1 | 1 | 1 | 1 | 1 |
| Did the report of the review contain an explicit statement that the review methods were established before the conduct of the review and did the report justify any significant deviations from the protocol? | 0 | 1 | 0 | 1 | 0 | 1 | 1 | 0 | 0 |
| Did the review authors explain their selection of the study designs for inclusion in the review? | 0 | 1 | 1 | 0 | 0 | 0 | 0 | 1 | 1 |
| Did the review authors use a comprehensive literature search strategy? | 1 | 0 | 1 | 1 | 0 | 1 | 1 | 0 | 1 |
| Did the review authors perform study selection in duplicate? | 0 | 0 | 1 | 0 | 0 | 1 | 1 | 1 | 0 |
| Did the review authors perform data extraction in duplicate? | 0 | 1 | 0 | 1 | 0 | 0 | 0 | 1 | 0 |
| Did the review authors provide a list of excluded studies and justify the exclusions? | 0 | 0 | 0 | 0 | 0 | 1 | 0 | 0 | 0 |
| Did the review authors describe the included studies in adequate detail? | 1 | 0 | 0 | 1 | 1 | 0 | 0 | 0 | 0 |
| Did the review authors use a satisfactory technique for assessing the risk of bias (RoB) in individual studies that were included in the review? | 1 | 0 | 0 | 1 | 0 | 1 | 1 | 1 | 1 |
| Did the review authors report on the sources of funding for the studies included in the review? | 0 | 0 | 0 | 0 | 0 | 0 | 0 | 0 | 0 |
| If meta-analysis was performed did the review authors use appropriate methods for statistical combination of results? | 1 | 1 | 1 | 1 | 1 |  | 1 | 1 | 0 |
| If meta-analysis was performed, did the review authors assess the potential impact of RoB in individual studies on the results of the meta-analysis or other evidence synthesis? | 1 | 0 | 0 | 1 | 0 |  | 0 | 0 | 0 |
| Did the review authors account for risk of bias in individual studies when interpreting/discussing the results of the review? | 1 | 0 | 0 | 1 | 0 | 1 | 1 | 0 | 1 |
| Did the review authors provide a satisfactory explanation for, and discussion of, any heterogeneity observed in the results of the review? | 1 | 1 | 0 | 1 | 0 | 1 | 1 | 0 | 1 |
| If they performed quantitative synthesis did the review authors carry out an adequate investigation of publication bias (small study bias) and discuss its likely impact on the results of the review? | 1 | 0 | 0 | 1 | 0 |  | 1 | 0 | 1 |
| Did the review authors report any potential sources of conflict of interest, including any funding they received for conducting the review? | 1 | 1 | 1 | 1 | 1 | 1 | 1 | 1 | 0 |
| Overall quality | Critically low | Critically low | Critically low | Low | Critically low | Moderate | Low | Critically low | Critically low |

| **Supplementary material S5: Results of the included systematic reviews according to outcomes (n = 9).** | | | | | | | | | | | | |  |
| --- | --- | --- | --- | --- | --- | --- | --- | --- | --- | --- | --- | --- | --- |
| **Study** | **Woolen (2022)** | **Berge (2017)** | **Huncharek (2007)** | | **Penninkilampi (2018)** | **Kadry Taher (2019)** | **Chang (2020)** | **Lewis (2023)** | **Finley (2017)** | **Boon (2024)** |  |  |  |
| Cosmetic use | | | | | | | | | | |  |  |  |
| Ovarian cancer | 11 studies Increase OR: 1.47; 95% CI: 1.31 to 1.65^a^ | 27 studies Increase RR: 1.22; 95% CI: 1.13 to 1.30^a^ | 27 studies No differences  RR: 1.03; 95% CI: 0.80 to 1.37^a^ | 56 studies Increase OR: 1.31; 95% CI: 1.24 to 1.39^a^ | | 27 studies  Increase OR: 1.28; 95% CI: 1.20 to 1.37 ^a^ |  |  |  |  | | | |
| Cervical cancer |  |  |  |  | |  |  |  |  | 1/1 studies reported no differences | | | |
| Uterine cancer |  |  |  |  | |  |  |  |  | 5/5 studies reported no differences | | | |
| Stomach cancer |  |  |  |  | |  |  |  |  | 1/2 studies reported no differences | | | |
| Occupational | | | | | | | | | | | |  |  |
| Stomach cancer |  |  |  | |  |  | 13 studies Increase RR: 1.21; 95% CI: 1.03 to 1.42^a^ |  |  |  | |  |  |
| Pleural cancer |  |  |  | |  |  |  | 1 study No differences  Male OR: 0.6; 95% CI: 0.1 to 4.3^a^ |  | 1/1 studies reported no differences | |  |  |
| Pleural mesthelioma |  |  |  | |  |  |  | 1 study No differences  Male OR: 0.74; 95% CI: 0.04 to 14.0^a^  1 study No differences  Female OR: 2.46; 95% CI: 0.42 to 14.3^a^ | 3 studies  No events reported |  | |  |  |
| Peritoneal cancer |  |  |  | |  |  |  | 1 study No differences  Male OR: 1.7; 95% CI: 0.7 to 4.1^a^ |  |  | |  |  |
| Peritoneal mesthelioma |  |  |  | |  |  |  | 1 study No differences  Male SIR: 1.06; 95% CI: 0.03 to 5.92^a^ |  |  | |  |  |
| Lung cancer |  |  |  | |  |  |  |  |  | 3/3 studies reported no differences | |  |  |
| Colorectal cancer |  |  |  | |  |  |  |  |  | 0/1 studies reported no differences | |  |  |
| Bladder cancer |  |  |  | |  |  |  |  |  | 1/1 studies reported no differences | |  |  |
| Kidney cancer |  |  |  | |  |  |  |  |  | 1/1 studies reported no differences | |  |  |
| Prostate cancer |  |  |  | |  |  |  |  |  | 1/1 studies reported no differences | |  |  |
| All type cancer |  |  |  | |  |  |  |  |  | 1/1 studies reported no differences | |  |  |
| CI: confidence interval; RR: risk ratio; OR: odds ratio; SIR: Standardized Incidence Ratio.  Statistically significant findings are shown in bold.  a. Effect size adjusted for confounders. | | | | | | | | | | | | |  |
